# Validation of an automated shape-matching algorithm for biplane radiographic spine osteokinematics and radiostereometric analysis error quantification

**DOI:** 10.1371/journal.pone.0228594

**Published:** 2020-02-14

**Authors:** Craig C. Kage, Mohsen Akbari-Shandiz, Mary H. Foltz, Rebekah L. Lawrence, Taycia L. Brandon, Nathaniel E. Helwig, Arin M. Ellingson

**Affiliations:** 1 Division of Rehabilitation Science, Department of Rehabilitation Medicine, University of Minnesota, Minneapolis, Minnesota, United States of America; 2 Rehabilitation Medicine Research Center, Department of Physical Medicine and Rehabilitation, Mayo Clinic, Rochester, Minnesota, United States of America; 3 Department of Biomedical Engineering, University of Minnesota, Minneapolis, Minnesota, United States of America; 4 Department of Psychology, University of Minnesota, Minneapolis, Minnesota, United States of America; 5 School of Statistics, University of Minnesota, Minneapolis, Minnesota, United States of America; 6 Division of Physical Therapy, Department of Rehabilitation Medicine, University of Minnesota, Minneapolis, Minnesota, United States of America; 7 Department of Orthopaedic Surgery, University of Minnesota, Minneapolis, Minnesota, United States of America; Texas A&M University, UNITED STATES

## Abstract

Biplane radiography and associated shape-matching provides non-invasive, dynamic, 3D osteo- and arthrokinematic analysis. Due to the complexity of data acquisition, each system should be validated for the anatomy of interest. The purpose of this study was to assess our system’s acquisition methods and validate a custom, automated 2D/3D shape-matching algorithm relative to radiostereometric analysis (RSA) for the cervical and lumbar spine. Additionally, two sources of RSA error were examined via a Monte Carlo simulation: 1) static bead centroid identification and 2) dynamic bead tracking error. Tantalum beads were implanted into a cadaver for RSA and cervical and lumbar spine flexion and lateral bending were passively simulated. A bead centroid identification reliability analysis was performed and a vertebral validation block was used to determine bead tracking accuracy. Our system’s overall root mean square error (RMSE) for the cervical spine ranged between 0.21–0.49mm and 0.42–1.80° and the lumbar spine ranged between 0.35–1.17mm and 0.49–1.06°. The RMSE associated with RSA ranged between 0.14–0.69mm and 0.96–2.33° for bead centroid identification and 0.25–1.19mm and 1.69–4.06° for dynamic bead tracking. The results of this study demonstrate our system’s ability to accurately quantify segmental spine motion. Additionally, RSA errors should be considered when interpreting biplane validation results.

## Introduction

Back pain is the most debilitating musculoskeletal impairment afflicting today’s society [1]. Annual low back pain prevalence has been estimated to be between 22–65% [2] and more recently, one-month prevalence has been estimated to be as high as 30.8% [3]. Neck pain is similarly pervasive, with annual prevalence reported to be between 30–50% in adults [4]. Low back pain and neck pain often progress into chronic conditions ranking first and fourth, respectively, in years lived with disability [1]. Standard clinical assessment of low back and neck pain is currently limited and typically includes a subjective interview, physical examination, and may include standard radiographs or advanced imaging such as computed tomography (CT) or magnetic resonance imaging (MRI) [5, 6]. Despite the increasing utilization of diagnostic imaging, evidence is immerging that these imaging techniques may only offer nominal insight into the mechanisms of spine pain [4, 5, 7–10]. This may be due to the limitation of current clinical imaging techniques that generally capture static, two-dimensional (2D) images of the spine; often in non-functional, non-weightbearing positions. Optical motion capture systems, which are commonly used in research, allow for the attainment of dynamic, functional, three-dimensional (3D) motion analysis. However, these systems are prone to skin/marker motion artifact, marker placement error, and are not capable of accurately determining the underlying osteokinematic motion [11, 12].

Biplane radiography overcomes these limitations and is capable of producing highly-accurate, segmental osteokinematics [13–18]. As such, biplane radiography captures functional, real-time bone motion and holds promise for advancing spine care from a diagnostic, prognostic, and treatment perspective. Although this developing technology holds great potential, several important steps must be addressed prior to utilizing such a system. Most biplane radiographic systems are custom-made with independently adjustable components and, therefore, require a validation process to ensure appropriate accuracy. This is especially important due to the potential of an increase of radiation exposure associated with radiographic imaging if inappropriate acquisitions are acquired. Additionally, radiographic techniques can vary greatly at different regions of the body; therefore, it is important to consider joint-specific validation to ensure maximum accuracy and minimization of radiation exposure.

The post-processing of biplane images to determine osteokinematics is known as 2D/3D shape-matching. This process requires hours of manual-alignment or semi-automated tracking of the bone model over the two radiographic projections and is a significant limitation of this data collection methodology. Therefore, developing an automated 2D/3D shape-matching algorithm is critical to utilizing biplane radiography. To-date, only a few labs have demonstrated the feasibility and validity of shape-matching at the spine [14, 16, 19–21].

Radiostereometric analysis (RSA), which involves the tracking of implantable tantalum beads within the bones of interest, has been widely used to validate biplane systems and shape-matching algorithms and is considered the “gold standard” for tracking osteokinematics [13–16, 19]. However, there is inherent error associated with bead centroid identification, both on the static CT images (i.e. static bead centroid identification error) and on the dynamic radiographic images during kinematic tracking (i.e. dynamic bead tracking error). Static bead centroid identification is sensitive to errors due to CT image distortion, potential artifact from adjacent implanted beads [16], and human error. Dynamic bead tracking errors result in additional error in the definition of bead-based coordinate systems, especially for closely approximated beads, which is a particular challenge at the spine due to the small size of the vertebrae. Further, dynamic bead tracking is sensitive to bead occlusion from adjacent beads, low-resolution images, and radiographic distortion. [22]. Although the accuracy of RSA has been well-documented using inter-bead distances [16, 19, 22, 23], this metric is not directly related to kinematic measures of interest (i.e. relative position and orientation). Further, the influences of static bead centroid identification and dynamic bead tracking errors have not been examined. Therefore, the practical functional accuracy of RSA is unclear. A Monte Carlo simulation approach offers a solution to quantify and understand these inherent errors. This is done by systematically varying the individual bead centroid locations and thus altering the resultant local coordinate systems–when applied to the anatomic coordinate system, this provides meaningful quantification of error for a specific setup.

Therefore, the purpose of this study was to verify our custom biplane video-radiography system’s data acquisition methods and validate our custom, automated 2D/3D shape-matching algorithm using CT bone models against RSA for cervical and lumbar spine kinematics in a cadaveric specimen. Additionally, this study examined two potential sources of error inherent within the current gold standard of RSA; static bead centroid identification and dynamic bead tracking error and applied these errors to our testing setup using a Monte Carlo simulation approach.

## Materials and methods

With approval from the University of Minnesota Anatomy Bequest Program (and associated informed consent), a fresh-frozen, cadaveric specimen (male, 55 years of age, 1.8 m, 70.3 kg) fully intact from the torso superiorly, was obtained from the University of Minnesota Anatomy Bequest Program. The specimen had no prior history of spine surgery, pathology, or metal implants. The specimen was screened for spinal range of motion (ROM) and appropriate anthropometrics to ensure safe specimen handling within the laboratory environment and appropriate sizing for the CT scanner.

### Procedures

#### Bead placement and bone models

The specimen was thawed at room temperature prior to bead placement and dynamic motion assessment. Four, 1.6 mm tantalum beads were surgically implanted into the cortical bone of vertebrae C4-C6 and L3-L4 for RSA [14–16, 19, 20]. An anterior approach was used for the cervical spine and a posterior approach for the lumbar spine. Beads locations were pre-drilled and beads were inserted with a spring-loaded injector and secured with liquid adhesive. The surgical sites were sutured to ensure stable bead placement and to minimize soft tissue disruption.

**Fig 1:**
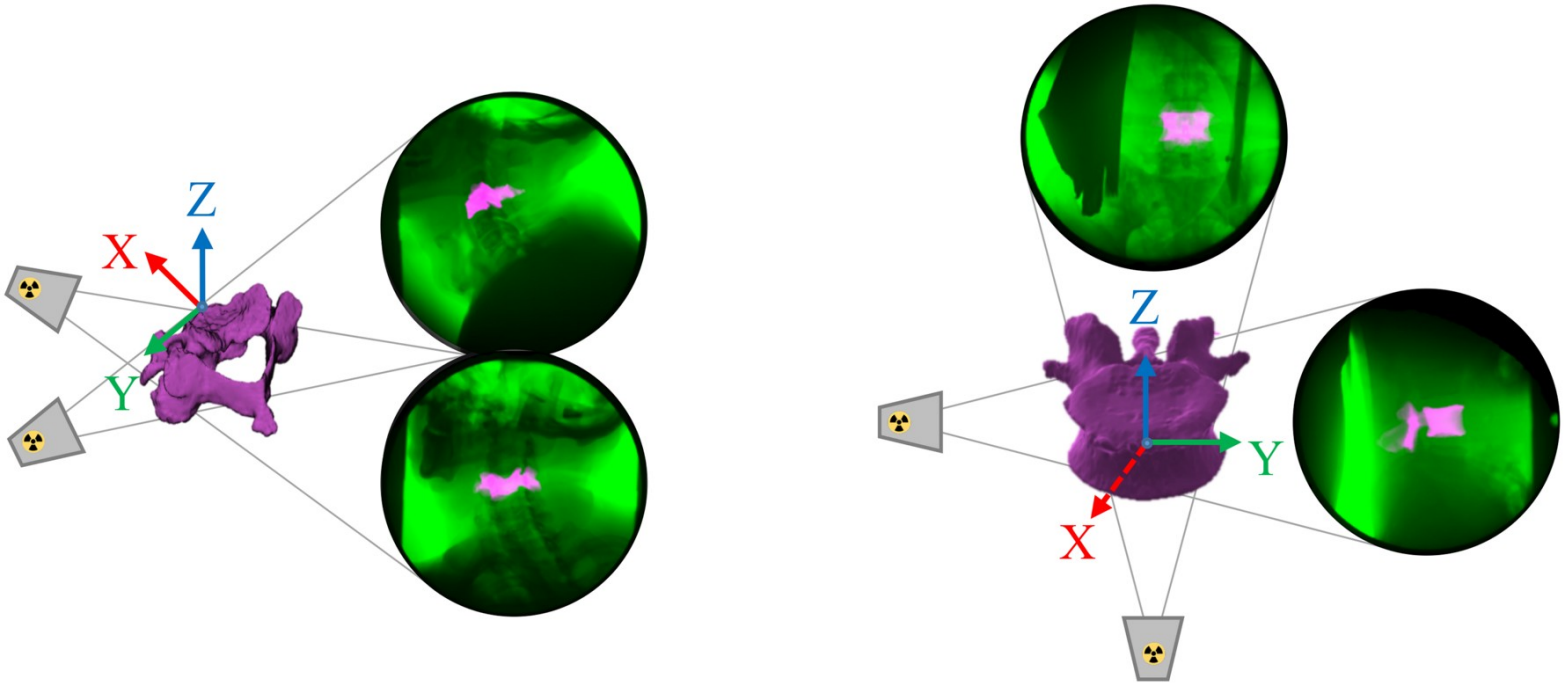
2D/3D shape-matching schematic. 2D/3D shape-matching with biplane radiographic images and associated digitally reconstructed radiographs (DRR’s) for *(Left)* cervical spine and *(Right)* lumbar spine with inset C4 and L4 CT bone models and local coordinate systems.

CT images were acquired following bead implantation (Siemens Somatom Sensation 64; Forchheim, Germany; 120 kVp; 0.23 × 0.23 × 0.60 mm for the cervical spine and 0.30 × 0.30 × 0.60 mm for the lumbar spine). Bone models were segmented from the CT scan for spinal levels C4-C6 and L3-L4 and bead centroids were identified (Mimics; Materialise, Plymouth, MI, USA). Each cervical spine bone model’s anatomic coordinate system was constructed by identifying the following anatomic landmarks: anterior/superior vertebral body and the most lateral/superior left and most lateral/superior right vertebral notches. Each lumbar spine bone model’s anatomic coordinate system was constructed by identifying the following landmarks: anterior/superior vertebral body, most lateral/superior vertebral body left and right. Each vertebrae was then assigned a local coordinate system from the anatomic landmarks applied to the anterior/superior vertebral body (Fig 1: X-axis = positive anteriorly, Y-axis = positive left, Z-axis = positive superiorly) [24]. Prior to 2D/3D shape-matching, beads in the CT were masked consistent with surrounding bone quality to avoid bias (ImageJ/Fiji; US NIH, Bethesda, MD, USA) [16].

#### Cervical spine dynamic cadaveric motion assessment

**Fig 2:**
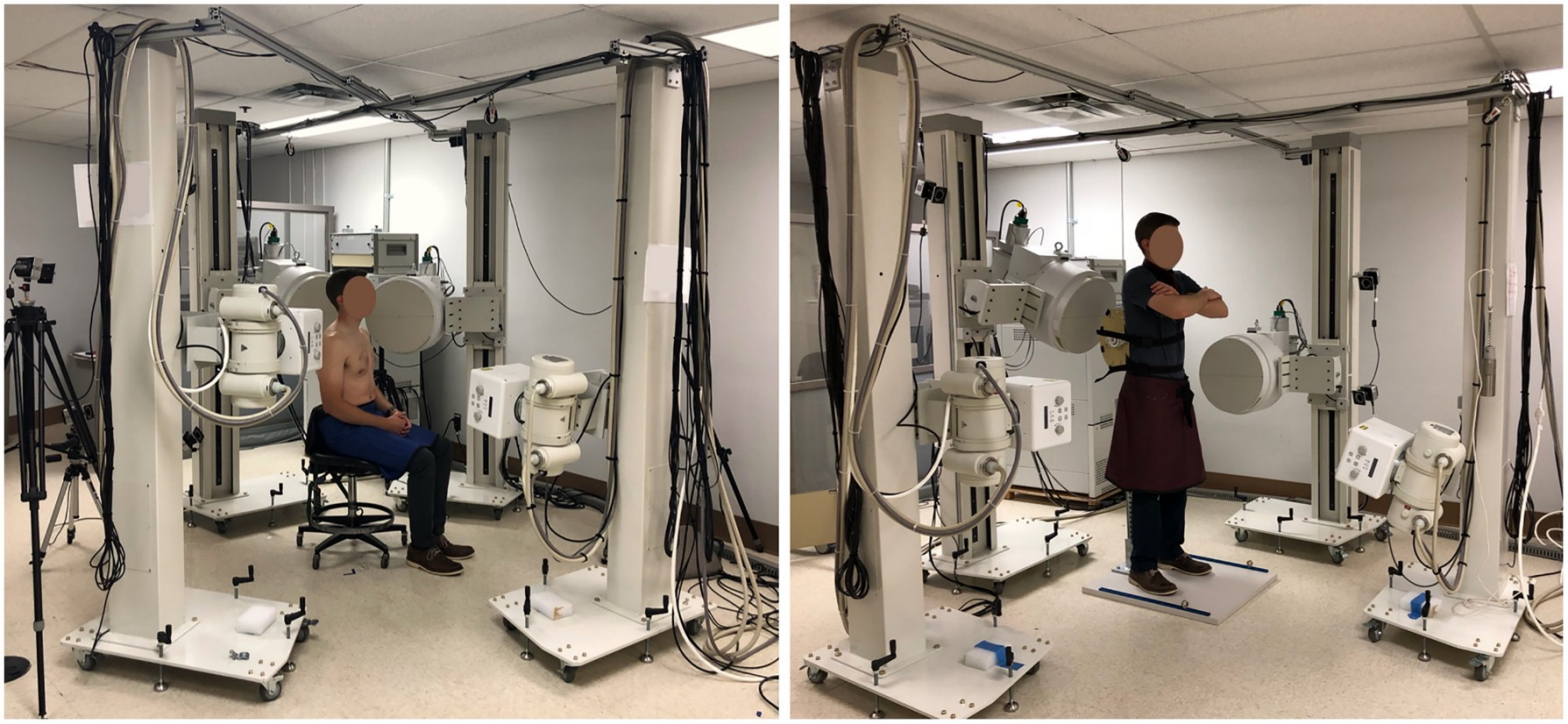
Biplane radiography setup. *(Left)* Cervical spine setup with two offset systems and interbeam angle of 55°, 16” image intensifiers and high-speed cameras. One system is oriented parallel to the horizontal and the other is oriented above the horizontal. *(Right)* Lumbar spine setup with attached attenuator and orthogonal systems. One system is oriented parallel to the horizontal and the other is oriented above the horizontal *Note*: *Validation was performed using a cadaveric specimen; however*, *the setup is demonstrated with a live subject*. *Lumbar acquisition was performed with the cadaveric specimen inverted and the AP system oriented 8° above the horizontal*.

Cervical spine motion data was collected using a custom biplane radiographic imaging system (Imaging Systems and Services, Inc.; Painesville, OH, USA) consisting of two high-speed cameras (Xcitex ProCapture, Woburn, MA, USA) and two 16-inch image intensifiers (Thales 9447 QX; North American Imaging, Aurora, OH, USA) (Fig 2). The specimen was secured to a height-adjustable chair in the biplane field of view. The images intensifiers were oriented with a 55° interbeam angle and with one system oriented in the medial-lateral direction, parallel to the horizontal and the second system in the anterior-oblique direction, 8° above the horizontal (Fig 2). Dynamic images were obtained with a radiographic wedge filter (Ferlic Filter Co., LLC; White Bear Lake, MN, USA) using the following technique: 70 kV, 250 mA, 3.5 ms, at 60 Hz and 152 cm source-to-image receptor-distance (SID) [14, 16]. Passive motion of the head was elicited by a long-handled apparatus for three trials of flexion (from an extended position) and lateral bending through the available ROM–the average trial time was 3.03 seconds. Images for biplane calibration and undistortion were acquired using a precision-machined (<0.025 mm) 64-bead calibration cube and undistortion grid (XMA Lab; Brown University, RI, USA) [25, 26].

#### Lumbar spine dynamic cadaveric motion assessment

The specimen was inverted and secured to a height adjustable chair in the biplane field of view. A custom-attenuator was affixed to the lumbar spine to reduce radiographic “wash-out” of the vertebrae [27]. The image intensifiers were oriented with a 90° interbeam angle with one system oriented in the medial-lateral direction, parallel to horizontal and the second system in the anterior-posterior direction, 8° above the horizontal (Fig 2). Radiographic images were obtained with the following technique: 78–82 kV, 630 and 200 mA (medial-lateral and anterior-posterior systems, respectively), 5.07 ms, at 60 Hz and 167 cm SID [27]. A radiographic, wedge filter was used for the flexion trials for the medial-lateral system and for both systems for the lateral bending trials. Passive motion of the pelvis was elicited by a long-handled apparatus and harness to simulate two trials of lumbar flexion (from an extended position) and lateral bending–the average trial time was 1.43 seconds. Calibration and undistortion were completed as described above.

#### Static bead centroid identification analysis

A reliability analysis was conducted to determine the consistency with which bead centroids could be identified on the CT scan. Five separate raters reviewed the cadaveric specimen’s CT multiplanar reconstructions in Mimics and identified the centroids of 12 beads (four beads per level: C4—C6) on three separate occasions.

#### Dynamic bead tracking validation

**Fig 3:**
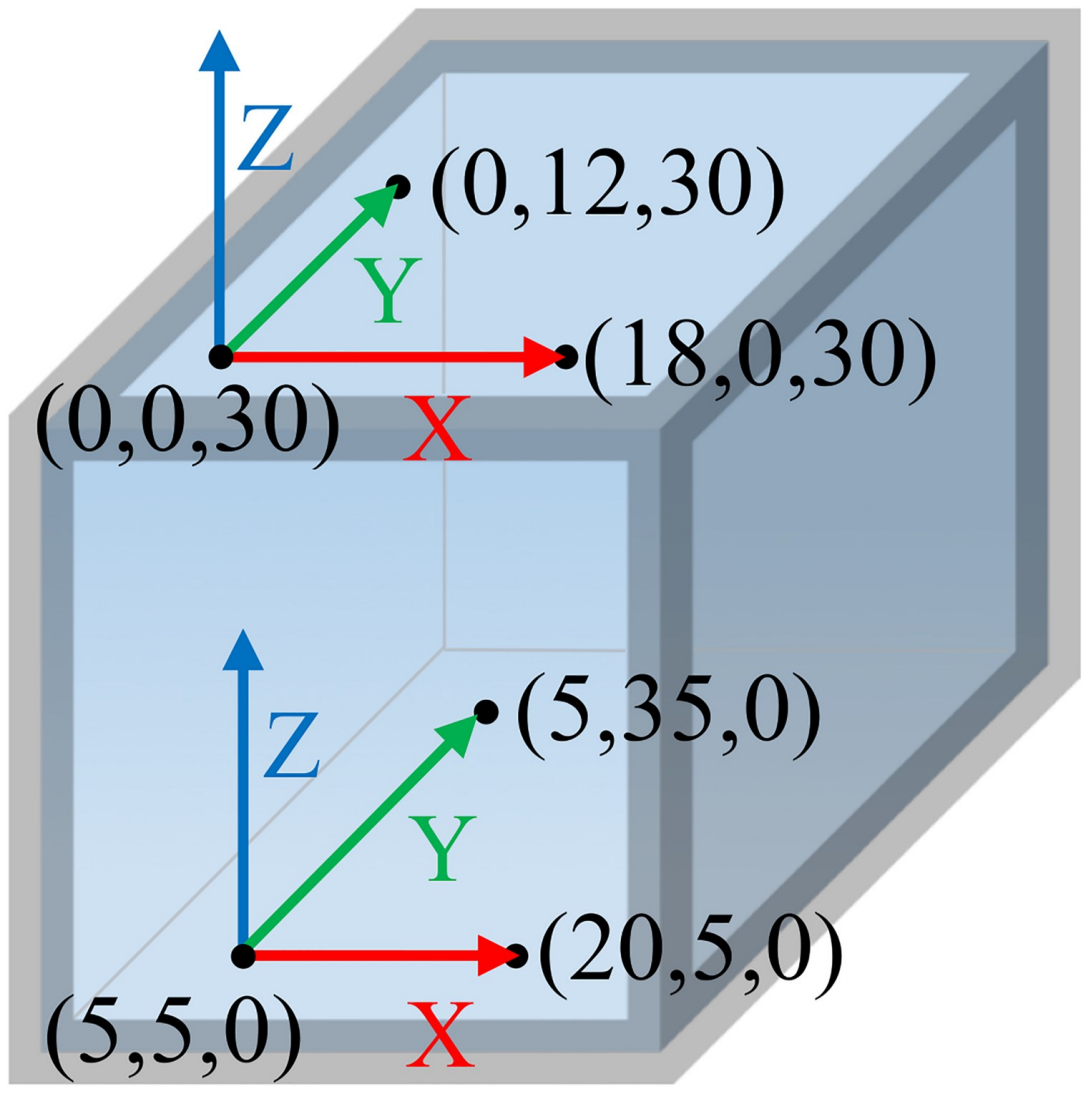
Validation block. Vertebral-sized, acrylic, validation block with tantalum beads and two associated coordinate systems. Dimensions/coordinates shown are in mm.

A custom, vertebral-sized, acrylic, validation block with six tantalum beads (1.6 mm) of known locations was precision-milled (<0.025 mm) to determine the error associated with dynamic bead tracking (Fig 3). From the known six beads coordinates, two orthogonal coordinate systems with known offsets were created (three beads for each coordinate system). The spacing of the beads within the validation block was chosen based on approximate vertebral RSA bead locations. This setup provides a more representative estimate of error than using only average inter-bead distances. The validation block was then moved through the biplane field of view to determine dynamic bead tracking error with the following technique: 50 kV, 63 mA, 3.57 ms, at 60 Hz and the same biplane setup as the cervical spine collection above. Bead centroids, and subsequently the offset between coordinate systems, were tracked throughout the dynamic trial and compared to the known coordinate system offset [28].

### Data analysis

A single initial bone location was manually placed using the graphical user interface in Autoscoper for each trial (Brown University, RI, USA). This position was input into a custom, automated 2D/3D shape-matching code to generate digitally reconstructed radiographs (DRR) using a standard ray casting approach (MATLAB, R2016B; The MathWorks, Inc.; Natick, MD, USA). A custom, automated 2D-3D image registration algorithm (Nelder-Mead Simplex optimization, maximizing a normalized cross-correlation similarity measure) was used to shape-match the DRR onto the biplane radiographic images [29]. The output of the 2D/3D shape-matching algorithm was *segmental* (C4, C5, C6 and L3, L4) and relative *intersegmental* (C4/C5, C5/C6, and L3/L4) position and orientation. Resulting bone positions and orientations were visualized in Autoscoper to qualitatively confirm appropriate shape-matching alignment of the DRR to the radiographic projections. Following visual inspection, the L3 level for each trial of lateral bending required additional manually-set frames beyond the single initial-frame to maximize alignment. A portion of one of these trials required manual shape-matching of six frames (out of 61 total analyzed frames) in Autoscoper, as it did not successfully auto-track with the algorithm.

Flexion trials were exported as a Y-X’-Z” sequence and lateral bending trials as a X-Z’-Y” sequence and filtered with a low-pass, fourth-order Butterworth filter with a cutoff frequency of 3 Hz in MATLAB (dynamic validation block tracking data was not filtered). A bead-based coordinate system was constructed for each vertebra and compared to the anatomic coordinate system to determine the transformation between the RSA and shape-matching output at each level. The output of the 2D/3D shape-matching algorithm was then compared directly to the RSA output for each frame of each trial. The overall output was reported across all trials and levels and each trial was weighted based on the number of frames of that trial. The ASTM International standards were used for reporting bias and precision when representing the differences between all frames of the 2D/3D shape-matching algorithm output and RSA output [30]. According to ASTM International, bias is “the difference between the expectation of the test results and an accepted reference value” and precision is “the closeness of agreement between independent test results obtained under stipulated conditions… expressed in terms of imprecision and computed as a standard deviation of the test results” [30]. For each direction of motion (flexion and lateral bending), the mean difference and the square root of the average variance and mean square error across all trials and levels was calculated to represent bias, precision, and Root Mean Square Error (RMSE), respectively. RMSE was calculated to represent the overall quality of agreement between the two methods.

*Monte Carlo Simulation*: Two Monte Carlo simulations were conducted to quantify how error in bead centroid digitization impacts the accuracy of RSA as a gold standard. Each Monte Carlo simulation was repeated 10,000 times to determine a stable estimate of errors. Two major components of error associated with RSA were considered: 1) error due to static bead centroid identification from CT and 2) error due to radiographic dynamic bead tracking. Both simulations randomly varied the bead centroid coordinates along all three axes (X, Y, Z) for all beads and at each cervical spine level (C4—C6). The degree of variation was determined through random sampling from one of two distributions: 1) errors due to static bead centroid identification were randomly sampled from a normal distribution defined from the bead centroid reliability data; zero was used as the mean of this distribution as the actual “absolute truth” location of the bead centroids was unknown; and 2) errors due to dynamic bead tracking were randomly sampled from a normal distribution defined using the bias and precision values from the validation block dynamic bead tracking data. New local coordinate systems were then created from these simulated bead coordinates and compared to the reference coordinate system, then transformed to the anatomic coordinate system to determine the translational and rotational error associated with RSA. For each simulation, the mean difference and the square root of the average variance and mean square error across levels (C4/C5, C5/C6) was calculated to represent bias, precision, and Root Mean Square Error (RMSE), respectively.

## Results

### Shape-matching vs. RSA

Mean bias, precision, and root mean square error (RMSE) of the kinematic differences between shape-matching and RSA across all spinal levels and trials for each motion at the segmental and intersegmental level are displayed in Tables 1 and 2.

**Table 1:** Cervical spine summary of means.

Flexion	Segmental			Intersegmental		
**Rotation (degrees)**	**LB (X)**	**FE (Y)**	**AR (Z)**	**LB (X)**	**FE (Y)**	**AR (Z)**
*Bias*	-0.49	0.35	0.48	-0.34	0.54	-0.23
*Precision*	0.36	0.57	0.35	0.50	0.80	0.39
*RMSE*	0.81	1.09	0.64	1.26	1.80	0.49
**Translation (mm)**	**AP (X)**	**ML (Y)**	**SI (Z)**	**AP (X)**	**ML (Y)**	**SI (Z)**
*Bias*	-0.46	0.10	-0.18	0.17	-0.24	-0.15
*Precision*	0.06	0.07	0.12	0.19	0.15	0.15
*RMSE*	0.48	0.23	0.24	0.42	0.28	0.34
**Lateral Bending**						
**Rotation (degrees)**	**LB (X)**	**FE (Y)**	**AR (Z)**	**LB (X)**	**FE (Y)**	**AR (Z)**
*Bias*	-0.15	-0.20	0.30	-0.11	0.64	-0.10
*Precision*	0.24	0.63	0.23	0.32	0.88	0.34
*RMSE*	0.33	1.16	0.50	0.42	1.44	0.76
**Translation (mm)**	**AP (X)**	**ML (Y)**	**SI (Z)**	**AP (X)**	**ML (Y)**	**SI (Z)**
*Bias*	-0.07	-0.25	-0.04	0.25	-0.11	0.08
*Precision*	0.07	0.08	0.18	0.26	0.15	0.25
*RMSE*	0.19	0.28	0.21	0.49	0.21	0.29

Bias, precision, and RMSE for all levels (C4-C6) and all motion trials for kinematic differences between shape-matching and RSA weighted by trial length. Lateral Bending (LB), Flexion/Extension (FE), Axial Rotation (AR), Anterior-Posterior (AP), Medial-Lateral (ML), Superior-Inferior (SI).

**Table 2:** Lumbar spine summary of means.

Flexion	Segmental			Intersegmental		
**Rotation (degrees)**	**LB (X)**	**FE (Y)**	**AR (Z)**	**LB (X)**	**FE (Y)**	**AR (Z)**
*Bias*	0.22	0.34	-0.05	0.35	0.73	0.26
*Precision*	0.23	0.25	0.31	0.40	0.31	0.42
*RMSE*	0.37	0.57	0.35	0.56	0.80	0.49
**Translation (mm)**	**AP (X)**	**ML (Y)**	**SI (Z)**	**AP (X)**	**ML (Y)**	**SI (Z)**
*Bias*	-0.57	0.30	-0.37	0.13	0.16	-0.90
*Precision*	0.22	0.35	0.30	0.27	0.53	0.34
*RMSE*	0.62	0.47	0.62	0.35	0.59	0.97
**Lateral Bending**						
**Rotation (degrees)**	**LB (X)**	**FE (Y)**	**AR (Z)**	**LB (X)**	**FE (Y)**	**AR (Z)**
*Bias*	0.22	0.27	-0.21	0.05	1.02	-0.34
*Precision*	0.50	0.22	0.44	0.54	0.29	0.62
*RMSE*	0.55	0.62	0.53	0.54	1.06	0.71
**Translation (mm)**	**AP (X)**	**ML (Y)**	**SI (Z)**	**AP (X)**	**ML (Y)**	**SI (Z)**
*Bias*	-0.50	0.10	-0.15	0.20	-0.20	-1.13
*Precision*	0.22	0.46	0.28	0.40	0.80	0.30
*RMSE*	0.55	0.50	0.63	0.45	0.83	1.17

Bias, precision, and RMSE for all levels (L3-L4) and all motion trials for kinematic differences between shape-matching and RSA weighted by trial length. Lateral Bending (LB), Flexion/Extension (FE), Axial Rotation (AR), Anterior-Posterior (AP), Medial-Lateral (ML), Superior-Inferior (SI).

#### Cervical spine (Table 1)

For flexion trials; mean RMSE was 0.32 mm and 0.85° for segmental motion, and 0.35 mm and 1.18° for intersegmental motion. This corresponded to a mean bias (± precision) of -0.18 mm (± 0.08 mm) and 0.11° (± 0.43°) for segmental motion, and -0.07 mm (± 0.16 mm) and -0.01° (± 0.56°) for intersegmental motion. For lateral bending trials, mean RMSE was 0.23 mm and 0.66° for segmental motion, and 0.33 mm and 0.87° for intersegmental motion. This corresponded to a mean bias (± precision) of -0.12 mm (± 0.11 mm) and -0.02° (± 0.37°) for segmental motion, and 0.07 mm (± 0.22 mm) and 0.14° (± 0.51°) for intersegmental motion. The mean total range of motion per trial across analyzed cervical spine levels and motions was 25.3° (± 8.8°), with a mean rotational velocity of 8.3 degrees/second (± 2.7 degrees/sec).

#### Lumbar spine (Table 2)

For flexion trials, mean RMSE was 0.57 mm and 0.43° for segmental motion, and 0.64 mm and 0.62° for intersegmental motion. This corresponded to a mean bias (± precision) of -0.21 mm (± 0.29 mm) and 0.17° (± 0.26°) for segmental motion, and -0.20 mm (± 0.38 mm) and 0.45° (± 0.38°) for intersegmental motion. For lateral bending trials, mean RMSE was 0.56 mm and 0.57° for segmental motion, and 0.82 mm and 0.77° for intersegmental motion. This corresponded to a mean bias (± precision) of -0.18 mm (± 0.32 mm) and 0.09° (± 0.39°) for segmental motion, and -0.38 mm (± 0.50 mm) and 0.24° (± 0.48°) for intersegmental motion. The mean total range of motion per trial across analyzed lumbar spine levels and motions was 24.5° (± 5.9°), with a mean rotational velocity of 19.6 degrees/second (± 9.4 degrees/sec).

#### Static bead centroid identification and dynamic bead tracking error

The results of the static bead centroid reliability assessment were averaged across the three trials for each rater. Interrater reliability was calculated as the standard error of the measurement (SEM) and was 0.12 mm. The results of the dynamic bead tracking analysis with the validation block are displayed in Table 3. The results of the Monte Carlo simulation for the error associated with the static bead centroid identification and dynamic bead tracking are found in Table 4. The overall range of RMSE associated with static bead centroid identification variability was 0.14–0.69 mm and 0.96–2.33°. The overall range of RMSE associated with dynamic tracking of the validation block was 0.25–1.19 mm and 1.69–4.06°.

**Table 3:** Validation block summary of means.

**Rotation (degrees)**	
*Bias*	0.03
*Precision*	0.49
*RMSE*	0.49
**Translation (mm)**	
*Bias*	0.03
*Precision*	0.21
*RMSE*	0.21

Summary of mean bias, precision, and root mean square error (RMSE) of the validation block dynamic bead tracking trial across all rotations and translations for tracking two orthogonal coordinate systems.

**Table 4:** Monte Carlo simulation results.

	Bead Centroid ID			Dynamic Bead Tracking		
**Rotation (degrees)**	**LB (X)**	**FE (Y)**	**AR (Z)**	**LB (X)**	**FE (Y)**	**AR (Z)**
*Bias*	0.00	0.01	0.01	0.00	0.00	0.03
*Precision*	1.05	2.33	0.96	1.83	4.06	1.69
*RMSE*	1.05	2.33	0.96	1.83	4.06	1.69
**Translation (mm)**	**AP (X)**	**ML (Y)**	**SI (Z)**	**AP (X)**	**ML (Y)**	**SI (Z)**
*Bias*	0.01	0.00	0.01	-0.02	0.00	0.04
*Precision*	0.69	0.35	0.14	1.19	0.62	0.25
*RMSE*	0.69	0.35	0.14	1.19	0.62	0.25

Results of a 10,000 sample Monte Carlo simulation for associated errors related to static bead centroid identification and validation block dynamic bead tracking. Summary of means: bias, precision, and RMSE across levels (C4/C5, C5/C6). Lateral Bending (LB), Flexion/Extension (FE), Axial Rotation (AR), Anterior-Posterior (AP), Medial-Lateral (ML), Superior-Inferior (SI).

## Discussion

The aim of this study was to validate our laboratory’s custom biplane radiography system and automated 2D/3D shape-matching algorithm relative to the gold standard RSA, in a cadaveric specimen; and estimate the magnitude of two sources of error associated with RSA. Our system’s overall RMSE at the cervical spine ranged between 0.21–0.49 mm and 0.42–1.80° and at the lumbar spine ranged between 0.35–1.17 mm and 0.49–1.06° for flexion and lateral bending motions. Results for both of these validations are reasonable given the results of previous works [14, 16, 20, 21], although differences in methods, analysis, and subjects make direct comparison challenging and limited.

*Anderst et al*. examined cervical spine 2D/3D shape-matching against RSA in human subjects undergoing cervical spine fusion and found an average segmental tracking precision of 0.19 mm for non-fused bones and an average intersegmental tracking precision of 0.4 mm and 1.1° for all bones including fused bones for flexion/extension and axial rotation [16]. *McDonald et al*. examined cervical spine bone model 2D/3D shape-matching against RSA in an ovine specimen for flexion/extension and axial rotation and found overall intersegmental translational bias to be within 0.56 mm, precision to be less than 0.15 mm, and RSME to be less than 0.56 mm [14]. In the same study, intersegmental rotational bias was found to be within 0.89°, precision to be less than 0.26°, and RSME to be less than 0.90° [14]. The present study found comparable values to *Anderst et al*.; however, both precision and RMSE values were higher than *McDonald et al*. Potential differences in bead placement, testing setup, and differences between human and ovine anatomy make the results difficult to compare directly, but may explain some of the difference between studies.

Previous lumbar spine 2D/3D shape-matching versus RSA validation works have been done by *Wu et al*. in a cadaveric model and *Dombrowski et al*. *in vivo* [20, 21]. *Wu et al*. examined an MRI bone model 2D/3D shape-matching at five positions throughout flexion and extension and found the average biplane shape-matching bias of a single segment/bone to be within 0.30 mm and 0.74° and precision to be within 0.39 mm and 0.83° across all planes of motion when compared to RSA [20]. *Dombrowski et al*. found precision values to between 0.2–0.3 mm for translation and 0.4–0.5° for rotation [21].

Cervical and lumbar spine accuracy measures for both motions were particularly sensitive to error about the flexion/extension axis. *Anderst et al*. had similar findings with an anterior approach for bead implantation at the cervical spine and a flexion/extension motion [16]; however, other validations have not examined lateral bending for which to compare the results herein. The high flexion/extension axis error was also found in the Monte Carlo simulation, suggesting some of the error attributed to shape-matching may be related to the anterior surgical approach, which resulted in placement of the RSA beads primarily along the flexion/extension axis. Overall, the results reported herein support our ability to continue into human subject collection at the cervical and lumbar spine.

**Fig 4:**
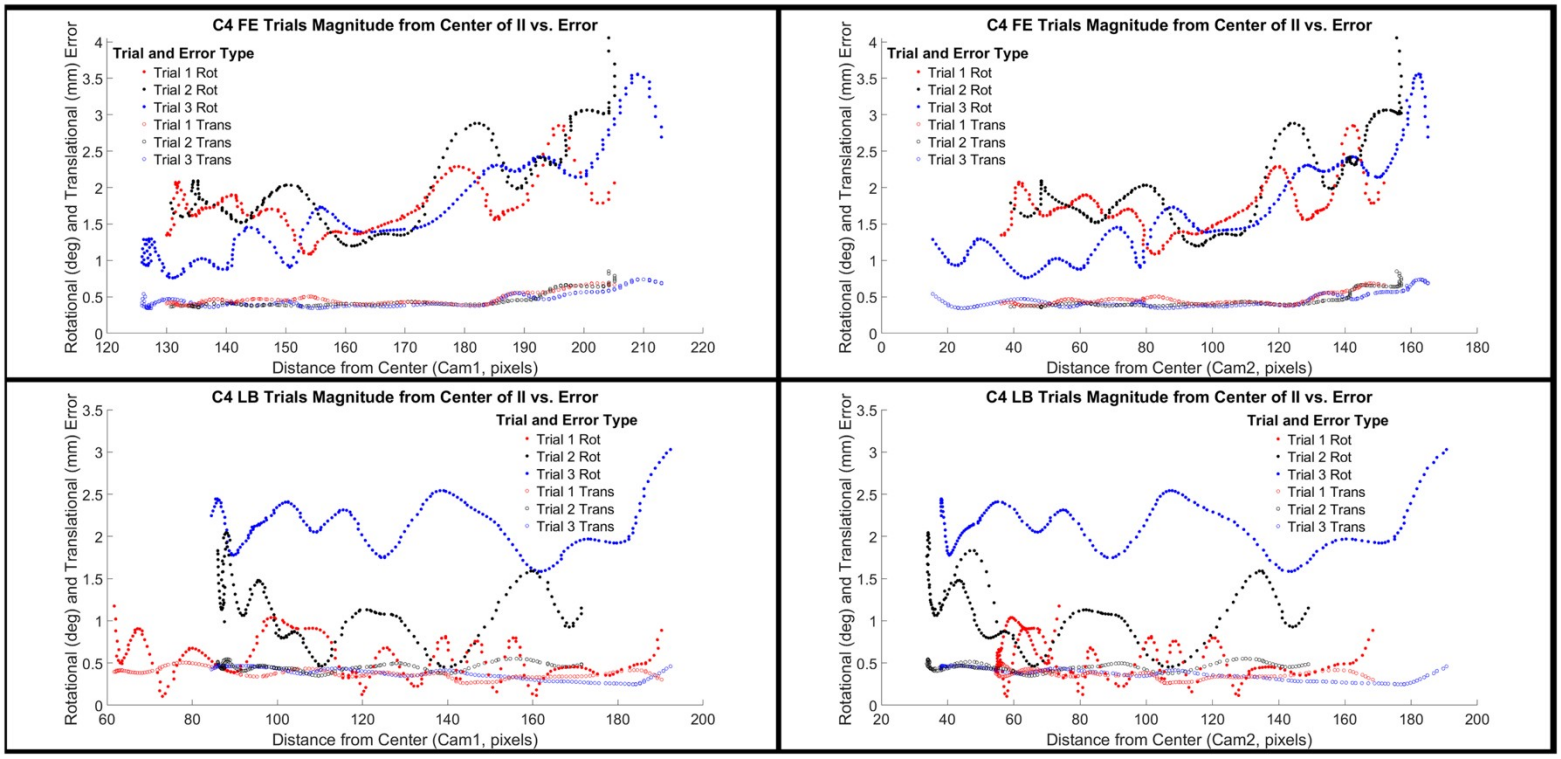
C4 distance from center of image intensifiers compared to tracking error. Overall rotational (degrees) and translational (mm) error magnitude is plotted against the distance of spinal level C4 relative to the center of the image intensifier (II) for each trial of flexion extension (FE) and lateral bending (LB) and each camera (1 and 2).

**Fig 5:**
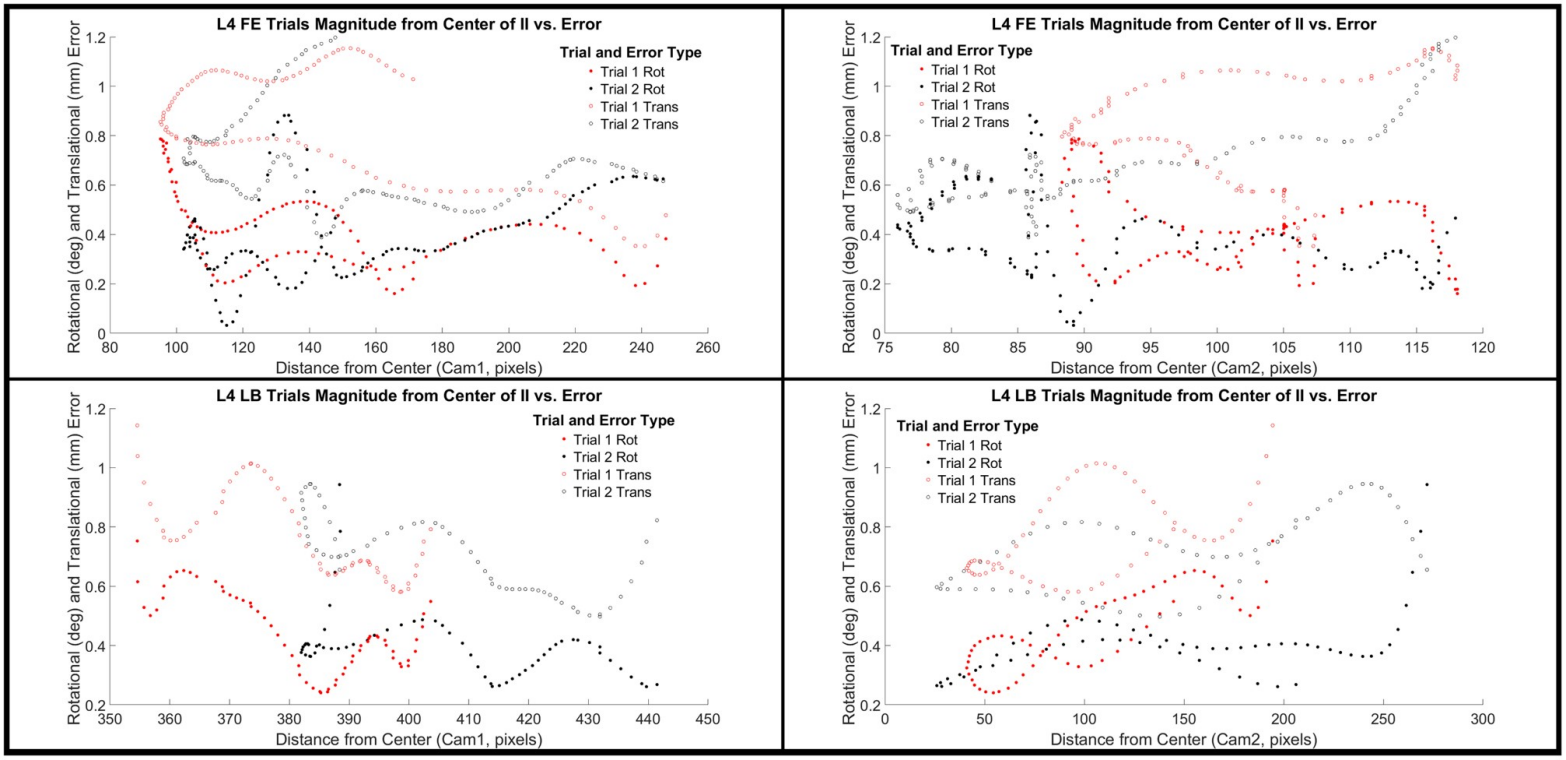
L4 distance from center of image intensifiers compared to tracking error. Overall rotational (degrees) and translational (mm) error magnitude is plotted against the distance of spinal level L4 relative to the center of the image intensifier (II) for each trial of flexion extension (FE) and lateral bending (LB) and each camera (1 and 2).

To better understand the error associated with edge effects from the image intensifier, we also examined the overall rotational and translational error magnitudes relative to the distance of the bone from the center of the image intensifiers for the cervical (Fig 4) and lumbar spine (Fig 5). We selected levels C4 and L4 as the representative levels for each motion type. From the review of the data in Figs 4 and 5, it is apparent that there are occasions when there does appear to be a trend of increasing error with greater distance from the center of the image intensifiers (Fig 4: Flexion/Extension trials 1–3 for rotational error in both Cam1 and Cam2); however, this does not appear to be a consistent trend amongst other trials. With that said, given the nature of radiographic imaging, it is still the best practice to seek to ensure that the region of interest remains as close as possible to the center of the image intensifiers during data collection.

The current study also found that the current gold standard for validation, RSA, may be sensitive to static bead centroid identification and dynamic bead tracking errors, which may result in RMSE values more than double what was found in the validation portion of this study. A previous study has reported an RSA precision of 0.09 mm at the cervical spine for implanted bead tracking [16]; however, as *Anderst et al*. notes, this is not an ideal gold standard as the precision of any reference should be “an order of magnitude better” than what it is being compared to [16]. Additionally, because RSA is dependent upon the same radiographic technology as shape-matching, both methods are prone to the same sources of imaging errors (e.g. calibration, undistortion). Precision values for the rigid validation block (0.21 mm) were higher than those found in previous studies tracking implanted beads (including 0.09 mm at the cervical spine [16], 0.06 mm in canine knees [22], 0.12 mm for tibiofemoral tracking [23], 0.18 mm for the lumbar spine [19]); however, these works only examined inter-bead distances, not the tracking of the origin of two coordinate systems, which is what is done for kinematic analysis. In the present study, the average of all inter-bead distances yielded a precision of 0.10 mm, which is comparable to previous studies. Bias for the rigid validation block dynamic tracking coordinate systems was 0.03 mm and the average of all inter-bead distances was 0.04 mm; comparable to previous studies which found no bias at the cervical spine [16], -0.02 mm in canine knees [22], and 0.08 mm for tibiofemoral tracking [23]. Therefore, the higher errors associated with the Monte Carlo simulation suggest the traditional method of establishing RSA validity (i.e. inter-bead distances) does not fully capture the errors associated with RSA.

This is the first study to the authors’ knowledge to specifically quantify the error associated with coordinate system bead tracking of RSA and bead centroid identification in relation to biplane radiography validation in the spine. When considering bead associated error the distances between the beads and the arrangement of the beads must also be considered, as closer bead approximations and linear arrangements will magnify angular centroid misidentification error. Therefore, RSA errors are likely greater in the spine due to the anatomical size. Although this study only examined sensitivity with 1.6 mm tantalum beads, it is likely that alternative sized beads will have different sensitivity for both dynamic and static errors.

The present study has limitations. One cadaveric specimen with a BMI of 21.7 was used for analysis with passive motion applied to the cervical and lumbar spine. The tracking algorithm is likely robust enough to track cervical spine kinematics in a larger individual, but tracking lumbar spine will be more challenging. Although soft tissues were left intact to best simulate *in vivo* conditions, it is understood that *in vivo* tissue quality and the effects of active/muscular motion generation are not necessarily represented. The beads were inserted into the anterior vertebral bodies at the cervical spine and the posterior elements of the lumbar spine due to the type of surgical approach. This resulted in close bead approximation, coronal plane arrangement, and possible magnification of error in generating the local coordinate systems. For the lumbar spine collection, the motion was carried out in an inverted position, which is not consistent with typical physiologic motion. Additionally, this validation work only examined imposed motion in two cardinal planes (no axial rotation) at spinal levels C4-C6 and L3-L4; therefore the validity of these methods in regards to other spinal levels, especially higher cervical levels due to occlusion from the mandible, cannot be determined from this present work. The custom 2D/3D shape-matching algorithm was not able to resolve the lumbar spine lateral bending trials from a single initial guess and required subsequent guesses, and in one particular case of an L3 lateral bending trial, required manual shape-matching for 10% of the frames of motion. This study examined two potential sources of RSA error reported in isolation; however, it should be noted that these two sources of error exist concurrently. Also, we did not examine another potential source of error associated with this process, which is the reliability of anatomic landmark identification. *Tashman et al*. examined anatomical landmark placement reliability and found the average standard deviation to be between 0.28–0.35 mm for femoral landmarks applied to bone models, which suggests a further level of variability when considering the overall error of this process [22]. Future validation studies may be worthwhile. Potential alternatives to RSA validation could include cortical bone pins or utilization of a material testing machine; however, these techniques are either limited to invasive studies or *ex vivo* collections [17]. Furthermore, these results suggest that 2D/3D shape-matching itself may potentially be of comparable accuracy to the traditional gold standard of RSA, as this method relies heavily on proper identification of the bead centroids rather than registration of an entire bone.

Biplane radiography allows for assessment of osteokinematic detail that is not possible with other technologies. Traditional imaging has 2D, static, or constrained space limitations, whereas biplane radiography has the versatility to be conducted in functional positions and dynamically with a high-rate of capture and 3D output. Despite these advantages, there are a number of limitations associated with radiographic technology that should be acknowledged. These include high initial cost and a significant level of experience and support required to operate the system. As this study demonstrates, there are potential challenges associated with validation to the current gold standard of RSA. Additional considerations including radiation exposure, multiple-step analysis, potential for summation of error, and limited field of view within the biplane system will need to be addressed in subsequent studies.

In summary, this study demonstrates the validity and accuracy of our laboratory’s biplane radiographic data acquisition and custom 2D/3D shape-matching algorithm; establishing the groundwork for our lab to proceed into *in vivo* applications at the spine. Furthermore, the potential limitations associated with the present gold standard (RSA) are analyzed in detail related to bead centroid identification and dynamic bead tracking error. These results suggest that the true accuracy of the 2D/3D shape-matching approach may be influenced by the underlying error associated with RSA and that these errors should be considered when interpreting biplane validation results.

## Abbreviations

CT: Computed tomography
MRI: Magnetic resonance imaging
RMSE: Root mean square error
ROM: Range of motion
RSA: Radiostereometric analysis
SID: Source-to-image receptor-distance
