# Correction: Validation of an automated shape-matching algorithm for biplane radiographic spine osteokinematics and radiostereometric analysis error quantification

**DOI:** 10.1371/journal.pone.0238274

**Published:** 2020-08-21

**Authors:** The PLOS ONE Staff

In the Funding statement, the author initials CK are missing from the funder the Foundation for Physical Therapy. The publisher apologizes for the error.

The correct Funding statement is: This work was supported by NIH/NICHD (National Institute of Child Health and Human Development): K12HD073945 (AE), F31HD087069 (RL), NIH/NIAMS (National Institute of Arthritis and Musculoskeletal and Skin Diseases) T32 AR050938, Musculoskeletal Training Grant (CK, RL), the Foundation for Physical Therapy (CK, RL), and the Minnesota Partnership for Biotechnology and Medical Genomics (MHP IF #14.02) (AE).
